# Comparative efficacy of pharmacological agents on reducing the risk of major adverse cardiovascular events in the hypertriglyceridemia population: a network meta-analysis

**DOI:** 10.1186/s13098-021-00626-7

**Published:** 2021-01-29

**Authors:** Yan-yan Qi, Li Yan, Zhong-min Wang, Xi Wang, Hua Meng, Wen-bo Li, Dong-chang Chen, Meng Li, Jun Liu, Song-tao An

**Affiliations:** 1 Department of Cardiology, People’s Hospital of Zhengzhou University No, 7, Weiwu Road, Zhengzhou, 450003 China; 2 Department of Cardiology, Hongxing hospital, Hami, 839000 China

**Keywords:** Hypertriglyceridemia, Major adverse cardiovascular events, Meta-analysis

## Abstract

**Background:**

Hypertriglyceridemia (HTG) is considered an independent risk factor for major adverse cardiovascular events (MACE).

**Methods:**

This study analyzed the effects of various agents on MACE risk reduction in HTG (serum triglyceride ≥ 150 mg/dl) populations by performing a network meta-analysis. We performed a frequentist network meta-analysis to conduct direct and indirect comparisons of interventions. PubMed, EMBASE, and the Cochrane library were searched for trials until Jul 6, 2020. Randomized controlled trials that reported MACE associated with agents in entire HTG populations or in subgroups were included. The primary outcome was MACE.

**Results:**

Of the 2005 articles screened, 21 trials including 56,471 patients were included in the analysis. The network meta-analysis results for MACE risk based on frequency data showed that eicosapentaenoic acid (EPA) (OR: 1.32; 95% CI 1.19–1.46), gemfibrozil (OR: 1.53; 95% CI 1.20–1.95), niacin plus clofibrate (OR: 2.00; 95% CI 1.23–3.25), pravastatin (OR: 1.32; 95% CI 1.15–1.52), simvastatin (OR: 2.38; 95% CI 1.55–3.66), and atorvastatin (OR: 0.55; 95% CI 0.37–0.82) significantly reduced the risk of MACE compared to the control conditions. In the subgroup analysis of HTG patients with triglycerides ≥ 200 mg/dL, bezafibrate (OR: 0.56; 95% CI 0.33–0.94), EPA (OR: 0.72; 95% CI 0.62–0.82), and pravastatin (OR: 1.33; 95% CI 1.01–1.75) significantly reduced the MACE risk.

**Conclusions:**

Simvastatin had a clear advantage in reducing the risk of MACE in the entire HTG population analyzed in this meta-analysis. EPA, but not omega-3 fatty acid, was considered an effective HTG intervention. Among fibrates, gemfibrozil was most effective, though bezafibrate may significantly reduce the risk of MACE in populations with triglyceride levels of 200–300 mg/dL.

*Trial registration* retrospectively registered in PROSPERO (CRD42020213705).

## Background

Hypertriglyceridemia (HTG), a condition in which triglyceride levels are elevated (> 150 mg/dl [1.69 mmol/L]), is considered an independent risk factor for major adverse cardiovascular events (MACE) [1, 2]. According to Task Force recommendations, people with mild or moderate HTG (triglycerides between 150 mg/dl [1.69 mmol/L] and 999 mg/dl [11.3 mmol/L]) have an increased risk of cardiovascular disease, and people with severe HTG (triglycerides of > 1000 mg/dl[11.3 mmol/L]) have an increased risk of acute pancreatitis [3–5]. Real-world data based on the CANHEART cohort showed that HTG was very common in the atherosclerotic cardiovascular disease population, and elevated triglycerides (TGs) increased the risk of arteriosclerotic cardiovascular diseases (ASCVDs) [6].

Serum TGs are the main components of chylomicrons and very low-density lipoproteins (VLDLs) [7, 8]. VLDLs and chylomicrons can permeate the arterial intima and selectively deposit, eventually causing the accumulation of cholesterol in the arterial intima and plaque formation [9]. In addition, elevated TGs have an important effect on coagulation and fibrinolysis, inhibiting fibrinolysis, increasing blood viscosity, and promoting thrombosis [10]. Therefore, HTG has a direct effect on atherosclerosis, increasing the risk of major cardiovascular events [11]. For mild to moderate HTG (177 mg/dl [2.0 mmol/L] to 885 mg/dl [10.0 mmol/L]), statins are considered the first-line drug. Although statins reduce low-density lipoprotein cholesterol (LDL-C) to a greater extent than they reduce TGs, a small reduction in TGs may further reduce the residual risk of cardiovascular disease, which indicates the cardiovascular risk among statin-treated individuals [12, 13]. In addition to statins, lifestyle modifications, such as cessation of alcohol consumption, reduced intake of rapidly metabolized carbohydrates, weight loss, and blood sugar control are highly effective ways to lower TG levels [14]. For many HTG patients, a further reduction in TGs may reduce the risk of residual cardiovascular disease [15]. Therefore, guidelines recommend fibrates, niacin, and omega-3 fatty acids if HTG persists despite the application of high-intensity statin therapy [14]

A previous meta-analysis showed that marine omega-3 fatty acids, especially high eicosapentaenoic acid (EPA)-content agents, have obvious TG-lowering effects; moreover, TG reduction was associated with major vascular event risk reduction [16]. Omega-3 fatty acids are generally well tolerated [17]. In a study of a human immunodeficiency virus (HIV)/acquired immunodeficiency syndrome (AIDS) population with HTG, the intake of EPA and docosahexaenoic acid (DHA) significantly reduced the risk of coronary heart disease (CHD) [18, 19]. It is believed that fibrates can reduce the risk of cardiovascular disease in high-risk cardiovascular disease populations, such as patients with HTG or with atherogenic dyslipidemia (triglycerides > 150 mg/dl [1.69 mmol/l] and high density lipoprotein cholesterol < 40 mg/dl [1.03 mmol/l] in men or < 50 mg/dl [1.29 mmol/l] in women)[20–22].

Currently, studies on the effects of various drugs on MACE outcomes in HTG populations are still insufficient; in many studies, HTG populations have been analyzed separately from major cardiovascular disease risk populations in only subgroup or post hoc analyses. This study will analyze the effects of various agents on reducing the MACE risk in HTG populations by a network meta-analysis to identify efficacious agents for clinical application.

## Methods

This network meta-analysis of published randomized controlled trials (RCTs) was performed according to the Preferred Reporting Items for Systematic Reviews and Meta-analyses incorporating Network Meta-Analyses (PRISMA-NMA) statement.

### Data sources, search strategy and selection criteria

Data from relevant studies were obtained by searching the PubMed, EMBASE, and Cochrane library databases without language restrictions. No initial date restriction was applied, and the search end date was Jul 6, 2020. Keywords included “hypertriglyceridemia”, “high triglyceride”, “high triacylglycerols”, “high triacylglycerides”, “survival”, “survivors”, “death”, “die”, “mortality”, “cardiovascular”, “MACE”, “random*”, “randomized”, and “randomized”. The search strategy details were list in Additional file 1: Table S1. Reference lists from identified trials and reviews were manually screened to identify additional trials.

### Inclusion and exclusion criteria

The inclusion criteria included the following: 1, a RCT design; 2, the inclusion of an HTG population; 3 a separate analysis of the HTG population in subgroup or post hoc analyses; and 3, the reporting of MACE-related outcomes. The exclusion criteria included the following: 1, studies without the inclusion of an HTG population; 2, studies that did not report the HTG population separately; 3, dosage-related studies; 4, lifestyle behavior or nursing-related intervention studies; or 5, studies that did not report MACE-related outcomes. Additionally, studies reporting zero MACE events in both groups were excluded because of small sample sizes or short follow-up periods. Conference summaries, editorial comments, and comments were also excluded.

### Data extraction and study quality assessment

Two authors independently extracted relevant information from the included studies. The extracted content included the name of the first author or collaborative organization, publication time, sample size, average age of the population, study abbreviations, intervention and control agents, outcome assessment, and follow-up period. MACE outcomes mainly included sudden cardiac death (defined as the unexpected death of an individual not attributable to an extracardiac cause, usually within one hour of symptom onset; it's a consequence of many cardiovascular conditions, and the leading cause of SCD is coronary heart disease (CHD), which accounts for over 70% of SCD cases) [23, 24], fatal/nonfatal myocardial infarction (defined as a condition where there is interruption of blood supply to a part of the heart that led to a fatal or severe nonfatal event), and nonfatal stroke (defined as non-convulsive loss of neurological function due to brain ischemia or intracranial hemorrhages that led to a fatal or severe nonfatal event). Individual study definitions were used for the outcome analysis (see Additional file 2: Table S2 for details). The potential risk of bias of the included RCTs was evaluated using the risk assessment tool recommended by the Cochrane Collaboration guidelines (assessing random sequence generation; allocation concealment; blinding of participants and personnel; blinding of outcome assessment; incomplete outcome data; selective reporting; and other risk) [25].

### Statistical analysis

Dichotomous data were combined to produce odds ratios (ORs), and 95% confidence intervals (CIs). Hazard ratios (HRs) and 95% CIs for Cox regression results were also calculated if raw event frequency data was not reported. A frequentist framework network meta-analysis was performed for mixed multiple treatment comparisons that combined direct and indirect evidence to obtain effect size [26]. Cochran’s Q test was used to assess homogeneity in the whole network and the homogeneity/consistency between designs. The pairwise comparison results based on the network meta-analysis were obtained, and the effective ranking of various agents was performed based on the P-score [27]. A comparison-adjusted funnel plot was used to assess funnel plot asymmetry for the network meta-analysis. A p value less than 0.05 was considered to indicate a significant difference. The analyses were performed using the “netmeta” package in R language (version 4.0.2).

## Results

### Literature screening

**Fig. 1:**
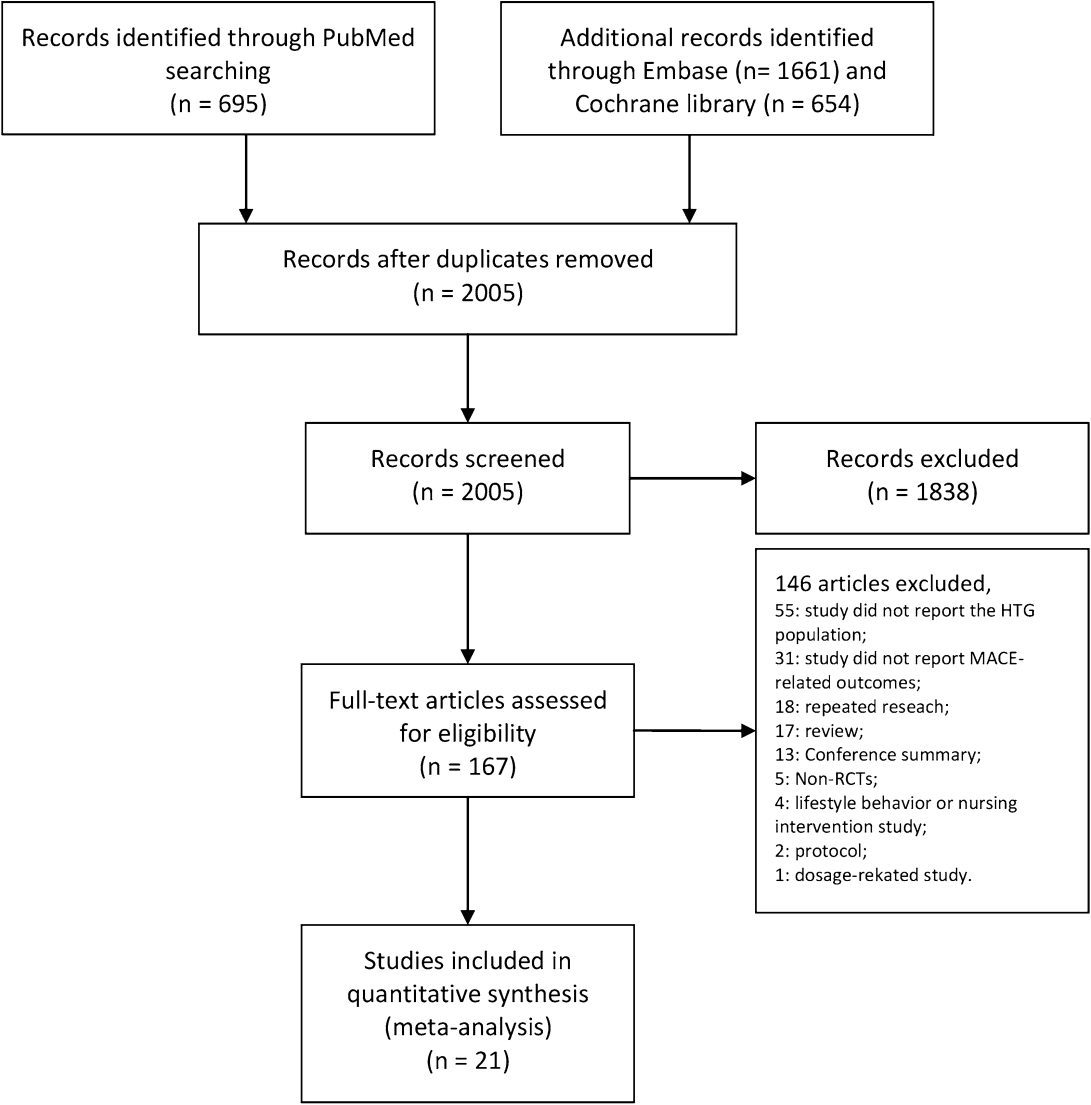
Flow chart illustrating the study selection process of this meta-analysis

Through public database searching, 695 studies were obtained from PubMed, 1661 studies were obtained from EMBASE, and 654 studies were obtained from the Cochrane library. After removing duplications, 2005 studies were obtained. A total of 1838 trials were excluded by title and abstract screening. A total of 167 full-text articles were reviewed. Additional studies were excluded on the basis of the following: the studies did not report the HTG population in their entirety or separately (55); the studies did not report MACE-related outcomes or reported no events in either arm (31); the studies were duplicated (18); the studies were reviews (17); the studies were conference summaries (13); the studies had non-RCT designs (5); the studies evaluated lifestyle behavior or nursing interventions (4); the studies were protocols (2); and the studies were dosage-related studies (1). For included post hoc studies, the latest reported results were included in the analysis. Finally, a total of 21 articles including 56,471 HTG patients were included in the analysis (Fig. 1) [28–48].

**Fig. 2:**
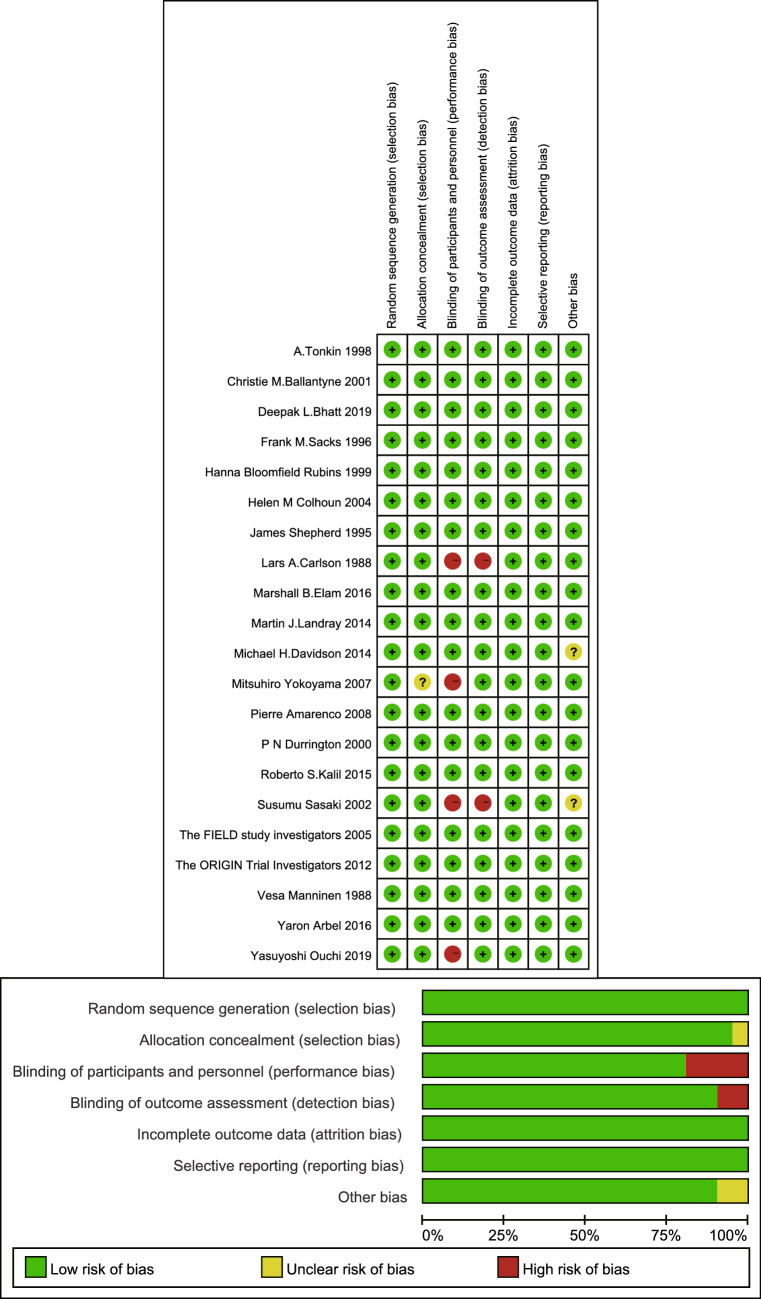
Risk of bias of each included study

Included studies were published from 1988 to 2019. In terms of sample size, with the exception of one study that included only 59 patients [42], the studies included more than 300 patients. The average age of the population was approximately 60 years old, except in one article in which the average age was approximately 80 years old [29]. The shortest follow-up period among the included studies was 1 year [42] (Table 1). In most studies, the most common MACE were cardiovascular death, nonfatal myocardial infarction (MI), nonfatal stroke, acute coronary syndrome, and coronary revascularization. However, there were studies that reported only death events [31, 34, 35, 42, 47] (Additional file 2: Table S2). Regarding the research quality assessment, only two articles did not state their blinding method [40, 47], and two articles performed only blinding of the assessors [29, 37]. The rest of the study designs were of high quality. Thus, the overall quality of included studies was relatively satisfactory (Fig. 2).

**Table 1:** The characteristics of included studies

Author	Year	Local	Sample size	Age# of intervention group	Age of Control group	Abbreviations	Intervention	Control	Follow-up
Bhatt [28]	2019	Multination	8179	64 (57–69)	64 (57–69)	REDUCE-IT	EPA	Placebo	4.9 Years
Ouchi [29]	2019	Japan	1046	80.6 ± 4.7	80.6 ± 4.7	EWTOPIA 75	Ezetimibe	Control	5 Years
Elam [30]	2016	US,Canada	3635	62.3 ± 6.8	62.3 ± 6.8	ACCORD	Fenofibrate	Placebo	4.7 Years
Arbel [31]	2016	Israel	458	58 ± 7	58 ± 7	BIP	Bezafibrate	Placebo	20 Years
Kalil [32]	2015	US	3413	70.8 ± 7.4	70.6 ± 7.2	AIM-HIGH	Niacin	Placebo	4.1 Years
Landray [33]	2014	UK,Scandinavia,China	6575	64.9 ± 7.5	64.9 ± 7.5	HPS2-THRIVE	Niacin plus laropiprant	Placebo	3.9 Years
Davidson [34]	2014	US	676	61 ()	61 (?)	FIRST	Fenofibrate	Placebo	108 Weeks
The ORIGIN Trial Investigators [35]	2012	Canada	4270	63.5 ± 7.8	63.6 ± 7.9	ORIGIN	Omega-3 fatty acid	Placebo	6.2 Years
Amarenco [36]	2008	Multinational	1575	63.0 ± 0.2	62.5 ± 0.2	SPARCL	Atorvastatin	Placebo	4.9 Years
Yokoyama [37]	2007	Japan	9211	61 ± 8	61 ± 9	JELIS	EPA	Control	5 Years
The FIELD study investigators [38]	2005	Multinations	5093	62.2 ± 6.9	62.2 ± 6.8	FIELD	Fenofibrate	Placebo	5 Years
Colhoun [39]	2004	UK, Ireland	1425	61.8 ± 8.0	61.5 ± 8.3	CARDS	Atorvastatin	Placebo	4 Years
Sasaki [40]	2002	Japan	497	55.5 ± 10.2	55.5 ± 10.1	NA	Pravastatin	Control	5 Years
Ballantyne [41]	2001	Multinations	458	57.7 ± 7.8	57.7 ± 7.8	4S	Simvastatin	Placebo	5.4 Years
Durrington [42]	2000	UK	59	55.2 ± 7.0	54.8 ± 10.2	NA	Omega-3 fatty acid	Placebo	1 Year
Rubins [43]	1999	US	1185	64 ± 7	64 ± 7	VA-HIT	Gemfibrozil	Placebo	5.1 Years
Tonkin [44]	1998	Australia, New Zealand	1490	62 (55–67)	62 (55–68)	LIPID	Pravastatin	Placebo	6.1 Years
Sacks [45]	1996	US, Canada	2079	59 ± 9	59 ± 9	CARE	Pravastatin	Placebo	5 Years
Shepherd [46]	1995	UK	3356	55.3 ± 5.5	55.1 ± 5.5	WSCPS	Pravastatin	Placebo	4.9 Years
Carlson [47]	1988	Sweden	301	Males 59.2 ± 0.4 Female 63.0 ± 0.7	Male 58.9 ± 0.4 Females 62.5 ± 0.9	SIHDSPS	Niacin plus Clofibrate	Control	5 Years
Manninen [48]	1988	Finland	1490	NA (40–55)	NA (40–55)	HHS	Gemfibrozil	Placebo	5 Years

*NA* not available, *EPA* eicosapentaenoic acid. The full name of abbreviation for each study and the detail of major adverse cardiovascular events assessment were list in Additional file 2: Table S2

^#^Average age showed as: Mean ± Standard deviation or median (minimum–maximum)

**Fig. 3:**
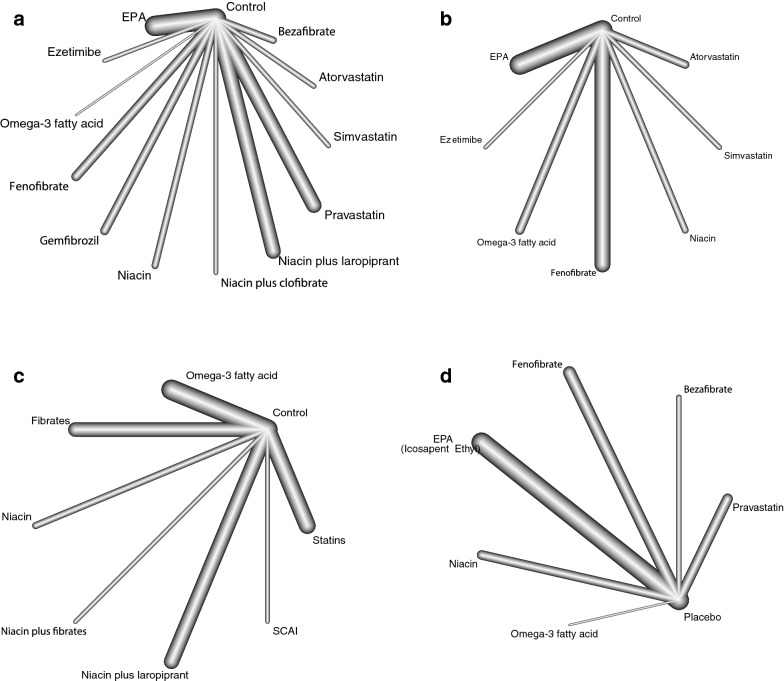
Network comparisons for the agents included in this analysis. **a** Analysis based on event frequency including various agents. **b** Analysis based on Cox regression results. **c** Analysis based on event frequency including classified agents. **d** Subgroup analysis of the moderate HTG population

**Fig. 4:**
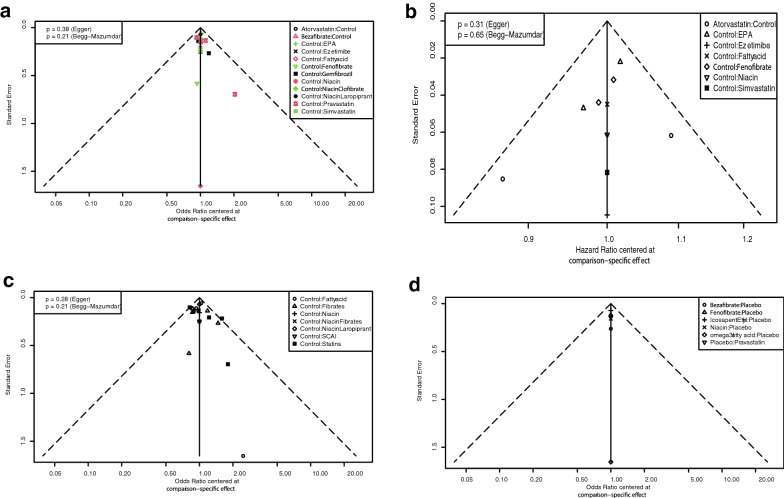
Comparison-adjusted funnel plot for assessing potential publication bias. **a** Analysis based on event frequency including various agents. **b** Analysis based on Cox regression results. **c** Analysis based on event frequency including classified agents. **d** Subgroup analysis of the moderate HTG population

The studies with MACE frequency data were analyzed first. The interventions included atorvastatin, bezafibrate, EPA, ezetimibe, omega-3 fatty acid, fenofibrate, gemfibrozil, niacin, niacin plus clofibrate, niacin plus laropiprant, pravastatin, simvastatin, and control (Fig. 3, A). Due to the included trials comparing interventions and control directly head to head, as well as the Q test results (p = 0.744), this study used consistency fixed-effect models. In pairwise comparisons, compared to control, EPA (OR: 1.32; 95% CI 1.19–1.46), gemfibrozil (OR: 1.53; 95% CI 1.20–1.95), niacin plus clofibrate (OR: 2.00; 95% CI 1.23–3.25), pravastatin (OR: 1.32; 95% CI 1.15–1.52), and simvastatin (OR: 2.38; 95% CI 1.55–3.66) could significantly reduce the risk of MACE. In addition, atorvastatin could also significantly reduce MACE risk compared to control (OR: 0.55; 95% CI 0.37–0.82) (Table 2). In omega-3 fatty acid research, due to the smaller number of patients and zero-events in the intervention arm [42], the accuracy of the omega-3 fatty acid results was low with a wide 95% CI. P-score ranking results showed that simvastatin (0.899), niacin plus clofibrate (0.812), and atorvastatin (0.767) have relative advantages. A comparison-adjusted funnel plot showed no publication bias (Fig. 4a).

**Table 2:** The league table for results based on event frequency estimates various agents according to their relative effects with odds ratio (95% confidence interval)

Atorvastatin (0.767)		*0.55 (0.37–0.82)*										
*0.59 (0.35–0.97)*	Bezafibrate (0.234)	0.94 (0.69–1.26)										
*0.55 (0.37–0.82)*	0.94 (0.69–1.26)	Control (0.120)	*1.32 (1.19*–*1.46)*	1.56 (0.95–2.56)	3.21 (0.13–82.07)	1.07 (0.86–1.34)	*1.53 (1.20*–*1.95)*	1.04 (0.76–1.41)	*2.00 (1.23*–*3.25)*	1.08 (0.94–1.24)	*1.32 (1.15*–*1.52)*	*2.38 (1.55*–*3.66)*
0.72 (0.48–1.10)	1.23 (0.90–1.69)	*1.32 (1.19*–*1.46)*	EPA (0.505)									
0.86 (0.45–1.62)	1.46 (0.82–2.61)	1.56 (0.95–2.56)	1.19 (0.72–1.96)	Ezetimibe (0.635)								
1.76 (0.07–46.16)	3.01 (0.12–77.90)	3.21 (0.13–82.07)	2.43 (0.10–62.34)	2.05 (0.08–54.52)	Omega-3 fatty acid (0.693)							
*0.59 (0.37–0.93)*	1.00 (0.69–1.45)	1.07 (0.86–1.34)	0.81 (0.63–1.04)	0.68 (0.40–1.18)	0.33 (0.01–8.58)	Fenofibrate (0.228)						
0.84 (0.52–1.35)	1.43 (0.98–2.10)	*1.53 (1.20*–*1.95)*	1.16 (0.89–1.51)	0.98 (0.56–1.70)	0.48 (0.02–12.29)	*1.43 (1.03*–*1.99)*	Gemfibrozil (0.656)					
*0.57 (0.34–0.94)*	0.97 (0.63–1.49)	1.04 (0.76–1.41)	0.78 (0.57–1.09)	0.66 (0.37–1.19)	0.32 (0.01–8.36)	0.97 (0.66–1.42)	0.68 (0.46–1.00)	Niacin (0.200)				
1.10 (0.58–2.06)	*1.87 (1.06*–*3.31)*	*2.00 (1.23*–*3.25)*	1.52 (0.93–2.49)	1.28 (0.64–2.56)	0.62 (0.02–16.52)	*1.87 (1.10*–*3.19)*	1.31 (0.76–2.25)	*1.93 (1.09*–*3.43)*	Niacin Clofibrate (0.812)			
*0.59 (0.39–0.91)*	1.01 (0.73–1.41)	1.08 (0.94–1.24)	*0.82 (0.69*–*0.97)*	0.69 (0.41–1.16)	0.34 (0.01–8.63)	1.01 (0.78–1.31)	*0.71 (0.54*–*0.93)*	1.04 (0.75–1.46)	*0.54 (0.33*–*0.89)*	Niacin Laropiprant (0.244)		
0.73 (0.47–1.11)	1.24 (0.89–1.72)	*1.32 (1.15*–*1.52)*	1.00 (0.85–1.19)	0.85 (0.51–1.41)	0.41 (0.02–10.56)	1.24 (0.95–1.61)	0.86 (0.66–1.14)	1.28 (0.91–1.79)	0.66 (0.40–1.09)	*1.22 (1.01*–*1.49)*	Pravastatin (0.509)	
1.31 (0.72–2.36)	*2.23 (1.32*–*3.76)*	*2.38 (1.55*–*3.66)*	*1.81 (1.16*–*2.81)*	1.53 (0.79–2.93)	0.74 (0.03–19.52)	*2.23 (1.38*–*3.61)*	1.56 (0.95–2.55)	*2.30 (1.36*–*3.90)*	1.19 (0.62–2.27)	*2.20 (1.41*–*3.46)*	*1.80 (1.15*–*2.82)*	Simvastatin (0.899)

*EPA* eicosapentaenoic acid

Italic font means statistical difference. Traditional pairwise at upper right side, network pairwise at lower left side

^#^The P-score is performed in brackets

This study also conducted an analysis based on the HR results of the Cox regression analysis as a supplement to the results of the event frequency analysis because several studies did not report event frequency results but did report HR results [35, 36, 38]. The interventions included atorvastatin, EPA, ezetimibe, omega-3 fatty acids, fenofibrate, niacin, simvastatin and controls (Fig. 3b). A consistency fixed-effect model was used in the analysis (Q test: p = 0.133). According to the pairwise results, EPA (HR: 1.12; 95% CI 1.08–1.17), simvastatin (HR: 1.38; 95% CI 1.17–1.61), and atorvastatin (HR: 0.90; 95% CI 0.82–0.99) significantly reduced the MACE risk (Table 3). P-score ranking showed that simvastatin (0.979), ezetimibe (0.739), and EPA (0.690) had relative advantages. A comparison-adjusted funnel plot showed no publication bias (Fig. 4b).

**Table 3:** The league table for results based on Cox-regression estimates various agents according to their relative effects with hazard ratio (95% confidence interval)

Atorvastatin (0.626)	*0.90 (0.82–0.99)*						
*0.90 (0.82–0.99)*	Control (0.159)	*1.12 (1.08*–*1.17)*	1.18 (0.96–1.45)	0.99 (0.90–1.08)	1.05 (0.99–1.10)	1.02 (0.90–1.15)	*1.38 (1.17*–*1.61)*
1.01 (0.91–1.12)	*1.12 (1.08*–*1.17)*	EPA (0.690)					
1.07 (0.85–*1.34)*	1.18 (0.96–1.45)	1.05 (0.85–1.30)	Ezetimibe (0.739)				
0.89 (0.78–1.01)	0.99 (0.90–1.08)	*0.88 (0.80*–*0.97)*	0.83 (0.67–1.04)	Omega-3 fatty acid (0.138)			
0.94 (0.84–1.05)	1.05 (0.99–1.10)	*0.93 (0.87*–*0.99)*	0.88 (0.72–1.09)	1.06 (0.96–1.17)	Fenofibrate (0.395)		
0.92 (0.79–1.07)	1.02 (0.90–1.15)	0.91 (0.80–1.03)	0.86 (0.68–1.09)	1.03 (0.89–1.20)	0.97 (0.85–1.11)	Niacin (0.275)	
*1.24 (1.03–1.50)*	*1.38 (1.17*–*1.61)*	*1.22 (1.04*–*1.44)*	1.16 (0.90–1.51)	*1.39 (1.16*–*1.67)*	*1.32 (1.11*–*1.56)*	*1.35 (1.11*–*1.65)*	Simvastatin (0.979)

*EPA* eicosapentaenoic acid

Italic font means statistical difference. Traditional pairwise at upper right side, network pairwise at lower left side

^#^The P-score is performed in brackets

We further classified the agents for analysis. The classifications included omega-3 fatty acids, fibrates, niacin, niacin plus fibrates, niacin plus laropiprant, selective cholesterol absorption inhibitors (SCAIs), and statins (Fig. 3c). Of these, statins are inhibitors of the hydroxymethylglutaryl-CoA (HMG-CoA) reductase enzyme to interfere with the endogenous synthesis of cholesterol. Ezetimibe inhibits the Niemann-Pick C1-Like 1 (NPC1L1) receptor to block intestinal cholesterol absorption. Omega-3 fatty acids are polyunsaturated fatty acids that regulate lipid metabolism by inhibiting lipogenesis and promoting lipolysis and are also involved in inflammatory regulation. Fibrates are a class of phenoxyisobutyric acid derivatives and serve as agonists for the peroxisome proliferator-activated receptor alpha (PPAR alpha) to limit triglyceride synthesis and promote lipoprotein lipase. Niacin binds with G protein-coupled receptor (GPR) 109A to reduce triglycerides and increase HDL-C. For this analysis, a consistency fixed-effect model was adopted (Q test: p = 0.098). According to the pairwise comparisons, omega-3 fatty acids (OR: 1.32; 95% CI 1.19–1.46), fibrates (OR: 1.21; 95% CI 1.05–1.40), niacin plus fibrates (OR: 2.00; 95% CI 1.23–3.25), and statins (OR: 1.44; 95% CI 1.27–1.63) significantly reduced the MACE risk compared to controls (Tables 4 and 5). A comparison-adjusted funnel plot showed no obvious publication bias (Fig. 4c).

**Table 4:** The league table for results based on event frequency estimates classified agents according to their relative effects with odds ratio (95% confidence interval)

Control (0.084)	*1.32 (1.19–1.46)*	*1.21 (1.05–1.40)*	1.04 (0.76–1.41)	*2.00 (1.23–3.25)*	*1.08 (0.94–1.24)*	*1.56 (0.95–2.56)*	*1.44 (1.27–1.63)*
*1.32 (1.19–1.46)*	Omega-3 fatty acid (0.600)						
*1.21 (1.05–1.40)*	0.92 (0.77–1.10)	Fibrates (0.444)					
1.04 (0.76–1.41)	0.78 (0.57–1.08)	0.85 (0.61–1.20)	Niacin (0.194)				
*2.00 (1.23–3.25)*	1.52 (0.93–2.48)	*1.65 (1.00*–*2.73)*	*1.93 (1.09*–*3.43)*	Niacin Fibrates (0.938)			
1.08 (0.94–1.24)	*0.82 (0.69*–*0.97)*	0.89 (0.73–1.09)	1.04 (0.75–1.46)	*0.54 (0.33*–*0.89)*	NiacinLaropiprant (0.242)		
1.56 (0.95–2.56)	1.18 (0.71–1.96)	1.29 (0.77–2.15)	1.51 (0.84–2.70)	0.78 (0.39–1.56)	1.45 (0.86–2.41)	SCAI (0.749)	
*1.44 (1.27–1.63)*	1.09 (0.93–1.28)	1.18 (0.98–1.43)	*1.39 (1.00*–*1.93)*	0.72 (0.44–1.18)	*1.33 (1.10*–*1.60)*	0.92 (0.55–1.53)	Statins (0.749)

*SCAI* selective cholesterol absorption inhibitors

Italic font means statistical difference. Traditional pairwise at upper right side, network pairwise at lower left side

^#^The P-score is performed in brackets

**Table 5:** The league table for results based on event frequency estimates agents for moderate HTG population according to their relative effects with odds ratio (95% confidence interval)

Bezafibrate (0.823)					0.56 (0.33–0.94)	
0.60 (0.34–1.05)	Fenofibrate (0.290)				0.93 (0.74–1.17)	
0.78 (0.46–1.33)	*1.30 (1.00*–*1.70)*	Icosapent Ethyl (0.675)			*0.72 (0.62*–*0.82)*	
0.58 (0.32–1.05)	0.97 (0.66–1.41)	0.74 (0.53–1.04)	Niacin (0.244)		0.97 (0.71–1.31)	
1.80 (0.07–47.78)	2.99 (0.12–77.15)	2.30 (0.09–58.97)	3.10 (0.12–80.45)	Omega-3 fatty acid (0.715)	0.31 (0.01–7.96)	
0.56 (0.33–0.94)	0.93 (0.74–1.17)	*0.72 (0.62*–*0.82)*	0.97 (0.71–1.31)	0.31 (0.01–7.96)	Placebo (0.161)	*1.33 (1.01*–*1.75)*
0.74 (0.41–1.33)	1.24 (0.86–1.77)	0.95 (0.70–1.29)	1.28 (0.85–1.94)	0.41 (0.02–10.68)	*1.33 (1.01*–*1.75)*	Pravastatin (0.593)

*HTG* hypertriglyceridemia

Italic font means statistical difference. Traditional pairwise at upper right side, network pairwise at lower left side

^#^The P-score is performed in brackets

Because of the different criteria for HTG diagnosis and interest in the treatment of moderate HTG patients, we performed a subgroup analysis of HTG patients with TGs ≥ 200 mg/dl (2.38 mmol/L). The interventions included EPA, fenofibrate, bezafibrate, niacin, omega-3 fatty acid, and pravastatin. The pairwise comparisons showed that bezafibrate (OR: 0.56; 95% CI 0.33–0.94), EPA (OR: 0.72; 95% CI 0.62–0.82), and pravastatin (OR: 1.33; 95% CI 1.01–1.75) significantly reduced the MACE risk. P-score ranking showed that bezafibrate (0.823), omega-3 fatty acid (0.715), and EPA (0.675) have relative advantages. No obvious asymmetry was found in the comparison-adjusted funnel plot.

## Discussion

TG level was an independent risk factor for cardiovascular events, despite adjustment for the LDL-C level [49]. Currently, there is a lack of meta-analyses of the effects of various agents on the MACE risk reduction in the HTG population. This study analyzed this topic by network meta-analysis. Statins, niacin, fibrates, omega-3 fatty acids, and ezetimibe were included in the analysis. It was found that simvastatin is superior to other agents for MACE risk reduction, and niacin plus clofibrate, EPA, and gemfibrozil also have relative advantages. The subgroup analysis of the population with TGs ≥ 200 mg/dL showed that bezafibrate and omega-3 fatty acids have relative advantages.

Niacin plus clofibrate has relative advantages in reducing the MACE risk in HTG patients based on a study published in 1988 [47]. Clofibrate is a fibric acid derivative used as a hypertriglyceridemia therapy [50]. However, clofibrate was withdrawn from the market in 2002 because of concern about its side effects, including elevated transaminase and rare acute liver injury [51]. In the analysis based on HR results, ezetimibe was found to reduce the risk of MACE, but the results were not significant. In the original study, ezetimibe reduced the risk of cardiovascular events in people with elevated LDL-C and those ≥ 75 years old. For those with LDL-C but without HTG, the effect of ezetimibe was obvious (HR: 0.65; 95% CI 0.47–0.91; p = 0.011) [29]. A systematic review also concluded that the combination of a statin and ezetimibe is more effective for lowering LDL-C levels than for lowering TG levels [52].

Two studies, the REDUCE-IT [28] and JELIS [37] studies, included EPA interventions. According to the results of these studies, EPA significantly reduced the risk of MACE compared to the control. However, omega-3 fatty acids showed no benefit in reducing the risk of MACE [35]. The results based on event frequency were also inaccurate due to zero events in the intervention arm [42]. However, it is generally believed that the effect of omega-3 fatty acids is inferior to that of EPA along, and DHA may cause an increase in LDL-C levels. This might be the reason why the Epanova (omega-3-carboxylic acids) study (NCT02104817) was discontinued; it had only limited benefits in mixed dyslipidemia patients [53]. In general, EPA is still considered an effective HTG treatment.

Fibrates including gemfibrozil, fenofibrate, and bezafibrate mainly act to clinically reduce TGs. Our results showed that gemfibrozil had a significant effect on reducing the risk of MACE compared with the control. However, its clinical application rate has gradually decreased, mainly due to muscle-related and blood-related side effects [54]. Therefore, gemfibrozil was mostly used for very high TG level intervention [55]. In the BIP study, bezafibrate was believed to be effective in those with TGs ≥ 200 mg/dL but not in those with TGs ≥ 150 mg/dL in the multivariate analysis after adjusting for age, previous MI, use of non-study-related lipid-lowering medication, and diabetes mellitus [31]. It is possible that in moderate HTG patients, bezafibrate has good TG-lowering effects that further reduce the MACE risk. A post hoc study also showed that among patients with TGs ≥ 200 mg/dL, those whose TGs decreased by > 0.5 mmol/L after intervention attained a greater effect in reducing their MACE risk [56]. However, compared with those in other studies, the patients in the BIP study had a lower TG upper limit (TG ≤ 300 mg/dL), so the benefit of bezafibrate in those with TGs ≥ 300 mg/dL is still unclear. Furthermore, the value of fenofibrate might have been under-estimated by population selection. In dyslipidemic diabetic patients, fenofibrate may be effective in reducing MACE risk [30, 38]. Pemafibrate is also a novel, highly selective peroxisome proliferator-activated receptor (PPAR)-α modulator (SPPARM) that can modulate lipid metabolism to decrease plasma triglyceride levels and increase high-density lipoprotein cholesterol levels[57, 58]. It has been approved in Japan for hyperlipidemia treatment [59]. However, MACE results for RCTs investigating pemafibrate were not completed at the initiation of this study, so pemafibrate was not analyzed in this work [60].

It should be noted that nonstatin intervention studies did not restrict the administration of statins in either arm. In some studies, all patients in both arms were treated with statins [32, 34]. Therefore, even if agents such as fibrates or EPA show benefits, the use of statins as first-line drugs will not change. Especially in comparisons with control groups, simvastatin showed obvious advantages.

In addition, familial hypertriglyceridemia (FHTG) is a familial autosomal dominant disease characterized by excessive triglyceride and VLDLs and is often found with polygenic genetic characteristics [61]. Due to insufficient investigation of the familial aggregation of HTG and the similar treatment strategy for reducing serum TG, there is still a lack of RCTs about FHTG. Thus, this work did not distinguish whether HTG in patients was familial or nonfamilial.

There were some limitations in this study. This study did not analyze the influence of patient characteristics and accompanying treatment on the results. Therefore, our study analyzed the effects of different agents in HTG populations. For patients with specific concomitant diseases, to account for accompanying treatment or type of dyslipidemia, more targeted studies are needed. In addition, this meta-analysis did not include studies analyzing lifestyle behavior interventions, although it is widely believed that good dietary and exercise habits are beneficial to TG reduction. Although the MACE criteria within individual studies were consistent, there were differences among the included studies. This study analyzed only the effects of various agents on reducing MACE risk in the HTG population, but side effects should be considered in clinical application when choosing personalized treatment regimens.

## Conclusions

Overall, among the statins, simvastatin maintained a clear advantage in reducing the risk of MACE in the HTG population. EPA, but not omega-3 fatty acid, was considered an effective HTG intervention. Among the fibrates, gemfibrozil was most effective, and bezafibrate may significantly reduce the risk of MACE in populations with TG levels of 200–300 mg/dL.

## Supplementary Information

**Additional file 1: Table S1.** Search strategy in PubMed database.

**Additional file 2: Table S2.** The full names of the included studies and details of the MACE assessments.

## Data Availability

All data generated or analysed during this study are included in this published article and its additional files.

## Abbreviations

AIDS: Acquired immunodeficiency syndrome
ASCVDs: Arteriosclerotic cardiovascular diseases
CHD: Coronary heart disease
CIs: Confidence intervals
DHA: Docosahexaenoic acid
EPA: Eicosapentaenoic acid
HIV: Human immunodeficiency virus
HTG: Hypertriglyceridemia
HRs: Hazard ratios
LDL-C: Low-density lipoprotein cholesterol
MACE: Major adverse cardiovascular event
MI: Myocardial infarction
OR: Odds ratio
PRISMA-NMA: Preferred reporting items for systematic reviews and meta-analyses incorporating network meta-analyses
RCT: Randomized controlled trial
SCAI: Selective cholesterol absorption inhibitor
TG: Triglyceride
VLDL: Very low-density lipoprotein
