# Supplementary material for: Comparative efficacy of pharmacological agents on reducing the risk of major adverse cardiovascular events in the hypertriglyceridemia population: a network meta-analysis
Source: Diabetol Metab Syndr. 2021 Jan 29;13:15. doi: 10.1186/s13098-021-00626-7 (PMC7845128; doi:10.1186/s13098-021-00626-7)
Supplement: Supplementary file 1 — Additional file 1: Table S1. Search strategy in PubMed database. [file 13098_2021_626_MOESM1_ESM.docx]

Supplementary table 1. Search strategy in PubMed database.

| Search number | Query | Search Details | Results |
| --- | --- | --- | --- |
| 4 | ((#1) AND (#2)) AND (#3) | ((((((((((((((((("mortality"[MeSH Subheading] OR "mortality"[All Fields]) OR "survival"[All Fields]) OR "survival"[MeSH Terms]) OR "survivability"[All Fields]) OR "survivable"[All Fields]) OR "survivals"[All Fields]) OR "survive"[All Fields]) OR "survived"[All Fields]) OR "survives"[All Fields]) OR "surviving"[All Fields]) OR ((("survivor s"[All Fields] OR "survivors"[MeSH Terms]) OR "survivors"[All Fields]) OR "survivor"[All Fields])) OR (("death"[MeSH Terms] OR "death"[All Fields]) OR "deaths"[All Fields])) OR "die"[All Fields]) OR ((("mortality"[MeSH Terms] OR "mortality"[All Fields]) OR "mortalities"[All Fields]) OR "mortality"[MeSH Subheading])) OR (((("cardiovascular system"[MeSH Terms] OR ("cardiovascular"[All Fields] AND "system"[All Fields])) OR "cardiovascular system"[All Fields]) OR "cardiovascular"[All Fields]) OR "cardiovasculars"[All Fields])) OR "Major Adverse Cardiovascular Events"[All Fields]) AND ((((((("hypertriglyceridaemia"[All Fields] OR "hypertriglyceridemia"[MeSH Terms]) OR "hypertriglyceridemia"[All Fields]) OR "hypertriglyceridaemias"[All Fields]) OR "hypertriglyceridemias"[All Fields]) OR "High triglyceride"[All Fields]) OR "High Triacylglycerols"[All Fields]) OR "High triacylglycerides"[All Fields])) AND (("random*"[All Fields] OR (((((((((((((((("random allocation"[MeSH Terms] OR ("random"[All Fields] AND "allocation"[All Fields])) OR "random allocation"[All Fields]) OR "random"[All Fields]) OR "randomization"[All Fields]) OR "randomized"[All Fields]) OR "randomisation"[All Fields]) OR "randomisations"[All Fields]) OR "randomise"[All Fields]) OR "randomised"[All Fields]) OR "randomising"[All Fields]) OR "randomizations"[All Fields]) OR "randomize"[All Fields]) OR "randomizes"[All Fields]) OR "randomizing"[All Fields]) OR "randomness"[All Fields]) OR "randoms"[All Fields])) OR (((((((((((((((("random allocation"[MeSH Terms] OR ("random"[All Fields] AND "allocation"[All Fields])) OR "random allocation"[All Fields]) OR "random"[All Fields]) OR "randomization"[All Fields]) OR "randomized"[All Fields]) OR "randomisation"[All Fields]) OR "randomisations"[All Fields]) OR "randomise"[All Fields]) OR "randomised"[All Fields]) OR "randomising"[All Fields]) OR "randomizations"[All Fields]) OR "randomize"[All Fields]) OR "randomizes"[All Fields]) OR "randomizing"[All Fields]) OR "randomness"[All Fields]) OR "randoms"[All Fields])) | 695 |
| 3 | ((random*) OR (randomized)) OR (randomised) | ("random*"[All Fields] OR (((((((((((((((("random allocation"[MeSH Terms] OR ("random"[All Fields] AND "allocation"[All Fields])) OR "random allocation"[All Fields]) OR "random"[All Fields]) OR "randomization"[All Fields]) OR "randomized"[All Fields]) OR "randomisation"[All Fields]) OR "randomisations"[All Fields]) OR "randomise"[All Fields]) OR "randomised"[All Fields]) OR "randomising"[All Fields]) OR "randomizations"[All Fields]) OR "randomize"[All Fields]) OR "randomizes"[All Fields]) OR "randomizing"[All Fields]) OR "randomness"[All Fields]) OR "randoms"[All Fields])) OR (((((((((((((((("random allocation"[MeSH Terms] OR ("random"[All Fields] AND "allocation"[All Fields])) OR "random allocation"[All Fields]) OR "random"[All Fields]) OR "randomization"[All Fields]) OR "randomized"[All Fields]) OR "randomisation"[All Fields]) OR "randomisations"[All Fields]) OR "randomise"[All Fields]) OR "randomised"[All Fields]) OR "randomising"[All Fields]) OR "randomizations"[All Fields]) OR "randomize"[All Fields]) OR "randomizes"[All Fields]) OR "randomizing"[All Fields]) OR "randomness"[All Fields]) OR "randoms"[All Fields]) | 1,380,077 |
| 2 | (((Hypertriglyceridemia) OR ("High triglyceride")) OR ("High Triacylglycerols")) OR ("High triacylglycerides") | "hypertriglyceridaemia"[All Fields] OR "hypertriglyceridemia"[MeSH Terms] OR "hypertriglyceridemia"[All Fields] OR "hypertriglyceridaemias"[All Fields] OR "hypertriglyceridemias"[All Fields] OR "High triglyceride"[All Fields] OR "High Triacylglycerols"[All Fields] OR "High triacylglycerides"[All Fields] | 16,845 |
| 1 | ((((((survival) OR (survivors)) OR (death)) OR (die)) OR (mortality)) OR (cardiovascular)) OR ("Major Adverse Cardiovascular Events") | ((((((((((((((("mortality"[MeSH Subheading] OR "mortality"[All Fields]) OR "survival"[All Fields]) OR "survival"[MeSH Terms]) OR "survivability"[All Fields]) OR "survivable"[All Fields]) OR "survivals"[All Fields]) OR "survive"[All Fields]) OR "survived"[All Fields]) OR "survives"[All Fields]) OR "surviving"[All Fields]) OR ((("survivor s"[All Fields] OR "survivors"[MeSH Terms]) OR "survivors"[All Fields]) OR "survivor"[All Fields])) OR (("death"[MeSH Terms] OR "death"[All Fields]) OR "deaths"[All Fields])) OR "die"[All Fields]) OR ((("mortality"[MeSH Terms] OR "mortality"[All Fields]) OR "mortalities"[All Fields]) OR "mortality"[MeSH Subheading])) OR (((("cardiovascular system"[MeSH Terms] OR ("cardiovascular"[All Fields] AND "system"[All Fields])) OR "cardiovascular system"[All Fields]) OR "cardiovascular"[All Fields]) OR "cardiovasculars"[All Fields])) OR "Major Adverse Cardiovascular Events"[All Fields] | 4,514,245 |
