# Supplementary material for: Comparative efficacy of pharmacological agents on reducing the risk of major adverse cardiovascular events in the hypertriglyceridemia population: a network meta-analysis
Source: Diabetol Metab Syndr. 2021 Jan 29;13:15. doi: 10.1186/s13098-021-00626-7 (PMC7845128; doi:10.1186/s13098-021-00626-7)
Supplement: Supplementary file 2 — Additional file 2: Table S2. The full names of the included studies and details of the MACE assessments. [file 13098_2021_626_MOESM2_ESM.docx]

Supplementary table 1. The full names of included studies and detail of MACE assessment.

| Author | Abbreviations | Full names | MACE assessment |
| --- | --- | --- | --- |
| Deepak L.Bhatt [29] | REDUCE-IT | the Reduction of Cardiovascular Events with Icosapent Ethyl–Intervention Trial | Cardiovascular death, nonfatal MI, nonfatal stroke, coronary revascularization, or unstable angina. |
| Yasuyoshi Ouchi [30] | EWTOPIA 75 | Ezetimibe Lipid-Lowering Trial on Prevention of Atherosclerotic Cardiovascular Disease in 75 or Older | Sudden cardiac death, fatal/nonfatal MI, ronary revascularization, or fatal/nonfatal stroke. |
| Marshall B.Elam [31] | ACCORD | Action to Control Cardiovascular Risk in Diabetes | Nonfatal MI, nonfatal stroke, or death from cardiovascular cause. |
| Yaron Arbel [32] | BIP | Bezafibrate Infarction Prevention | All-cause mortality |
| Roberto S.Kalil [33] | AIM-HIGH | The Atherothrombosis Intervention in Metabolic syndrome with low HDL/high triglycerides: Impact on Global Health outcomes | CHD death, nonfatal MI, ischemic stroke, hospitalization for acute coronary syndrome, or symptom-driven coronary or cerebral revascularization |
| Martin J.Landray [34] | HPS2-THRIVE | The Heart Protection Study 2–Treatment of HDL to Reduce the Incidence of Vascular Events | Nonfatal MI, death from coronary causes, stroke, or arterial revascularization |
| Michael H.Davidson [35] | FIRST | Evaluation of Choline Fenofibrate (ABT-335) on cIMT in Subjects with Type IIb Dyslipidemia with Residual Risk in Addition to Atorvastatin Therapy | Cardiovascular mortality |
| The ORIGIN Trial Investigators [36] | ORIGIN | the Outcome Reduction with an Initial Glargine Intervention | Death from cardiovascular causes |
| Pierre Amarenco [37] | SPARCL | The Stroke Prevention by Aggressive Reduction in Cholesterol Levels | Fatal and nonfatal stroke |
| Mitsuhiro Yokoyama [38] | JELIS | The Japan EPA Lipid Intervention Study | Sudden cardiac death, fatal and non-fatal MI, and other nonfatal events including unstable angina pectoris, angioplasty, stenting, or coronary artery bypass grafting |
| The FIELD study investigators [39] | FIELD | Fenofibrate Intervention and Event Lowering in Diabetes | Coronary heart disease death or non-fatal myocardial infarction. |
| Helen M Colhoun [40] | CARDS | Collaborative Atorvastatin Diabetes Study | Acute coronary heart disease events, coronary revascularisation procedures, or stroke. |
| Susumu Sasaki [41] | NA | NA | Fatal or non-fatal major coronary events, angina pectoris, MI, fatal or non-fatal stroke, and total mortality. |
| Christie M.Ballantyne [42] | 4S | Scandinavian Simvastatin Survival Study | Coronary death, nonfatal acute MI, resuscitated cardiac arrest, or definite silent MI. |
| P N Durrington [43] | NA | NA | Death from acute myocardial infarction |
| Hanna Bloomfield Rubins [44] | VA-HIT | the Veterans Affairs Cooperative Studies Program High-Density Lipoprotein Cholesterol Intervention Trial | Nonfatal myocardial infarction or death from coronary heart disease |
| A.Tonkin [45] | LIPID | Long-Term Intervention with Pravastatin in Ischaemic Disease (LIPID) | Death due to CHD and nonfatal myocardial infarction |
| Frank M.Sacks [46] | CARE | Cholesterol and Recurrent Events | Death from coronary heart disease or a symptomatic nonfatal MI. |
| James Shepherd [47] | WSCPS | West of Scotland Coronary Prevention Study | Nonfatal MI, death from coronary heart disease |
| Lars A.Carlson [48] | SIHDSPS | Stockholm Ischaemic Heart Disease Secondary Prevention Study | Death from Ischaemic Heart Disease |
| Vesa Manninen [49] | HHS | Helsinki Heart Study | Fatal and nonfatal MI, cardiac death |

Abbreviations: MI: myocardial infarction; NA: not available.
